# Supplementary material for: WordCommentsAnalyzer: A windows software tool for qualitative research
Source: F1000Res. 2018 Sep 4;7:536. Originally published 2018 May 3. [Version 2] doi: 10.12688/f1000research.14819.2 (PMC6137411; doi:10.12688/f1000research.14819.2)
Supplement: Supplementary file 3 [file f1000research-7-17614-s0002.tgz › b2a79496-4ada-47c4-9682-a99ce867836d.zip!/Eight Iranian Health Journals/The Journal of Toloo-e-Behdasht_25.docx]

© The Journal of Toloo-e-Behdasht. This work is licensed under CC BY 4.0 (https://creativecommons.org/licenses/by/4.0/).

Volume & Issue: دوره 13 - شماره 2

Title: بررسی کارایی نانو‌ذرات Fe3O4 اصلاح شده با پوست پرتقال در حذف یون‌های سرب و مس از محیط‌های آبی

http://tbj.ssu.ac.ir/article-1-1013-fa.html

مقدمه: ورود فلزات سنگین به منابع آب و زنجیره‌ی غذایی تهدید جدی برای سلامت انسان است. هدف از این تحقیق بررسی کارایی نانو‌ذرات Fe3O4 اصلاح شده با پوست پرتقال در حذف یون های سرب و مس از محیط‌های آبی است. روش بررسی: پژوهش تجربی و در سال 1391 انجام شد و از نانو‌ذرات Fe3O4 که با پوست پرتقال اصلاح گردید بعنوان جاذب استفاده شد. آزمون ها تحت فرایند ناپیوسته توسط 300 میلی‌گرم بر لیتر از جاذب با تغییر فاکتورهای مؤثر مانند زمان تماس (20، 40، 60، 80، 100، 150، 200 و 250 دقیقه)،pH (12-3)، دما (20، 30، 40 و 0C 50) و غلظت‌های 10 و 20 میلی‌گرم بر لیتر از یون‌های سرب و مس انجام گردید. سینتیک جذب و پارامترهای ترمودینامیک مطابق با شرایط تئوری ارزیابی شدند. یافته‌ها: بیشترین ظرفیت جذب یون‌های سرب و مس بر روی جاذب تحت شرایط بهینه (زمان تماس 40 دقیقه،pH برابر 6، غلظت mg/L20 و دمای 0C50) به ترتیب برابر mg/g06/70 و 21/71 حاصل شد. جذب یون‌های سرب و مس مطابق با مدل سینتیکی شبه درجه دو بود و به ترتیب با ضرایب همبستگی 91/0 و 99/0 با مدل جذب لانگمیر تناسب داشتند. مقادیر مثبت آنتالپی (∆Ho) طبیعت گرماگیر و مقادیر منفی انرژی آزاد (∆Go) خودبه خودی بودن فرایند را نشان داد. نتیجه‌گیری: در شرایط مطلوب 98 % جذب یون‌های سرب و مس بر روی جاذب اتفاق افتاد. نتایج نشان داد که نانو‌ذرات Fe3O4 اصلاح شده با پوست پرتقال می تواند جاذبی مؤثر برای حذف یون‌های سرب و مس از منابع آبی آلوده باشد. واژه های کلیدی: مس، سرب، نانو‌ذرات Fe3O4، پوست پرتقال، جذب

Title: بررسی عوامل تعیین کننده استفاده از نخ دندان بر مبنای مدل فرانظریه ای در دانش آموزان مقطع دبیرستان شهرستان پاکدشت سال 1391

http://tbj.ssu.ac.ir/article-1-1014-fa.html

مقدمه: با استفاده از مسواک همواره نقاطی از نواحی اینتر پروگزیمال قابل دسترس نمی باشد و حذف پلاک از این نواحی با استفاده از نخ دندان لازم است. هدف این مطالعه بررسی عوامل تعیین کننده بر رفتار استفاده از نخ دندان در دانش آموزان دبیرستانی براساس الگوی فرا نظریه ای می باشد. روش بررسی: مطالعه حاضر، مطالعه ای توصیفی- تحلیلی به روش مقطعی می باشد که با حضور 653 نفر از دانش آموزان دبیرستانی شهرستان پاکدشت که به روش نمونه گیری خوشه ای مرحله ای انتخاب شدند، در اردیبهشت سال 1391انجام شد. پرسشنامه محقق ساخته شامل اطلاعات زمینه ای، مراحل تغییر، خودکارآمدی، موانع و مزایای درک شده جهت جمع آوری داده ها بود. روایی محتوایی با استفاده از جدول لاوشه و97/0CVI= و پایایی آن با استفاده از روش آلفای کرونباخ (83/0) تایید شد. داده ها با نرم افزار 15 SPSSو آزمونهای logistic Regression spearman Correlation ، nonparametric tests ,ANOVAs مورد تجزیه و تحلیل قرار گرفتند. یافته ها: اکثریت دانش آموزان با73 درصد (477 نفر)، در استفاده از نخ دندان در مرحله پیش تفکر و تفکر قرار داشتند. نمره خودکارآمدی و مزایای درک شده آزمودنیها در مراحل اولیه تغییر رفتار پایین تر و موانع درک شده در مراحل اولیه بالاتر از مراحل نهایی بود (000/0 p<). همچنین افزایش میزان خودکارآمدی درک شده، شانس اقدام به استفاده از نخ دندان و نگهداری این رفتار به مدت بیشتر از 6 ماه را افزایش می دهد و افزایش در موانع درک شده افراد این شانس را کاهش می دهد (000/0 p<). نتیجه گیری: درصد بالایی از دانش آموزان جهت استفاده از نخ دندان در مرحله پیش تفکر و تفکر قرار داشتند، که احتمالا در آینده در معرض پوسیدگی بین دندانی قرار خواهند گرفت. برمبنای الگوی فرانظریه ای خودکارآمدی درک شده، ‌مزایا و موانع درک شده از عوامل تعیین کننده استفاده از نخ دندان می باشند که دربرنامه ریزی های آموزشی باید مد نظر قرار گیرند. واژه های کلیدی: نخ دندان، مدلهای آموزشی، دانش آموزان

Title: بررسی اثر مکمل یاری با ویتامین D بر شاخص های آنتروپومتریک در افراد مبتلا به سندرم متابولیک

http://tbj.ssu.ac.ir/article-1-1016-fa.html

مقدمه: چاقی در سراسر دنیا به‌ صورت اپیدمی درآمده که آن را به چالش سلامت عمومی و بالینی عظیمی تبدیل کرده است. یکی از اختلالات مرتبط با چاقی سندرم متابولیک می‌باشد. شواهد رو به افزایش نشان می‌دهند که سطح 25 – هیدروکسی ویتامین D3 ، ارتباط معکوس با چاقی و شاخص‌های آنتروپومتریک دارد. هدف مطالعه حاضر بررسی اثر مکمل‌ یاری با ویتامین D بر شاخص‌های آنتروپومتریک در افراد مبتلا به سندرم متابولیک می باشد. روش بررسی: طراحی این مطالعه به صورت کارآزمایی بالینی تصادفی شده دو سو کور بود. افراد شرکت کننده در مطالعه 88 زن و مرد سنین 30 تا 65 سال مبتلا به سندرم متابولیک، مراجعه کننده به کلینیک دیابت بیمارستان امام خمینی تهران بودند. 300هزار واحد بین المللی ویتامین D به صورت 6 پرل 50 هزار واحدی در گروه درمان و 6 پرل دارو نما حاوی پارافین خوراکی در افراد گروه کنترل در هفته اول مطالعه مصرف شد. سطوح (OH)D325 پلاسما و شاخص‌های آنتروپومتریک شامل وزن، نمایه توده بدنی، دور کمر و نسبت دور کمر به با سن در ابتدا و 3 ماه پس از شروع مطالعه اندازه‌گیری شد. یافته‌ها: سطوح (OH)D325 سرم افزایش معناداری از 20 نانوگرم در میلی‌لیتر در ابتدای مطالعه به 33 نانوگرم در میلی‌لیتر پس از 3 ماه مداخله در گروه درمان نشان داد(001/0P <). مقدار میانگین اندازه دور کمر در گروه درمان نسبت به ابتدای مطالعه کاهش معناداری نشان داد (005/0P =). پس از گذشت 3 ماه مکمل‌یاری با ویتامین D، در سایر شاخص‌های آنتروپومتریک گروه‌های مطالعه نیز تغییر معناداری مشاهده نشد. نتیجه گیری: مکمل‌یاری با ویتامین D به صورت روزانه 50 هزار واحد بین المللی به مدت 6 روز در افراد مبتلا به سندرم متابولیک که سطح سرمی ویتامین D پایین دارند پس از 3 ماه می تواند موجب افزایش سطح ویتامین سرم تا محدوده مطلوب وکاهش دور کمر شود اما سایر شاخص های آنتروپومتریک در افراد مبتلا به سندرم متابولیک را کاهش نمی دهد. این مقاله برگرفته از پایان نامه دانشجویی دانشگاه علوم پزشکی شهید صدوقی یزد می باشد.

Title: بررسی کارایی فرایند اکسیداسیون فنتون در حذف فنل از فاضلاب سنتتیک

http://tbj.ssu.ac.ir/article-1-1605-fa.html

مقدمه: فنل یکی از هیدروکربن های آروماتیک است که در پساب صنایعی مانند ساخت مواد شیمیایی، پالایشگاه نفت و پتروشیمی وجود دارد. سمیت بالا و اثرات سوء آن بر سلامتی موجودات زنده، دلیلی بر ضرورت کنترل غلظت این آلاینده در طبیعت است. هدف اصلی این مطالعه، بررسی کارایی فرایند اکسیداسیون فنتون در حذف فنل از فاضلاب سنتتیک است. روش بررسی: این تحقیق یک مطالعه تجربی می باشد. در مقیاس آزمایشگاهی و در سیستم بسته، اثر pH، مقادیر بهینه Fe2+ و H2O2، زمان تماس و غلظت های مختلف فنل بر راندمان حذف آن بررسی شد. فنل در نمونه های آزمایش توسط دستگاه کروماتوگرافی مایع با کارکرد بالا، مجهز به آشکارساز UV-Visible سنجش شد. یافته‌ها: نتایج نشان دادند، فنل با غلظت اولیه mM/L 6/1 در شرایط بهینه 3pH=، mM/L 09/0=Fe2+، mM/L 9/5= H2O2 (015/0 Fe2+/H2O2=) بعد از مدت زمان 15 دقیقه، حدود 97 % حذف شد. افزایش غلظت اولیه فنل از 6/1 به 7/3 mM/L، موجب کاهش راندمان حذف از 97 به 75 % شد. نسبت بهینه 01/0Fe2+/H2O2/phenol= بود. نتیجه گیری: در نتیجه، این روش می تواند به عنوان گزینه ای مناسب در حذف فنل جهت دستیابی به استانداردهای زیست محیطی مد نظر قرار گیرد.

Title: برآورد بار مالی حوادث ناشی از کار بر سازمان تأمین اجتماعی در استان یزد در سال های 88-1387

http://tbj.ssu.ac.ir/article-1-1017-fa.html

مقدمه: حوادث ناشی از کار هزینه های زیادی را بر اقتصاد ملی کشور تحمیل می کند. برآورد این هزینه ها به اتخاذ تصمیمات مناسب برای کنترل آنها کمک می کند. هدف اصلی این پژوهش برآورد خسارات اقتصادی ناشی از حوادث شغلی بر سازمان تأمین اجتماعی استان یزد در سال های 88-1387 می باشد. روش بررسی: این پژوهش یک مطالعه توصیفی مقطعی است که در آن هزینه های بیمه ای و درمانی ناشی از کل 990 حادثه ثبت شده طی سا لهای 1387و1388 در استان یزد تعیین شده است. گردآوری داده ها از طریق مصاحبه و بررسی مستندات موجود در اداره کل و مدیریت درمان تأمین اجتماعی یزد صورت گرفته است. داده های جمع آوری شده توسط نرم افزار 15 SPSS و با استفاده از آزمون های correlation و kruskal-wallis مورد تجزیه و تحلیل قرار گرفت. یافته ها: نتایج نشان داد که کل هزینه های درمانی و بیمه ای وارده ناشی از 990 مورد حادثه شغلی در دو سال مورد مطالعه معادل 54 میلیارد ریال بود در حالیکه که کل حق بیمه دریافتی از حادثه دیدگان طی دو سال یاد شده معادل 37/9 میلیارد ریال بوده است. نتیجه گیری: با توجه به وجود رابطه معنی دار بین نوع شغل و نوع کارگاه با هزینه های درمانی و بیمه ای، یافته های این مطالعه بر ضرورت سرمایه گذاری سازمان تأمین اجتماعی در زمینه ارتقاء ایمنی مشاغل و کارگاه های یاد شده تأکید می کند.

Title: بررسی آگاهی، نگرش و عملکرد مادران باردار در خصوص مصرف شیر و لبنیات در شهر یزد

http://tbj.ssu.ac.ir/article-1-1018-fa.html

مقدمه:شیر و فرآورده های آن با دارا بودن موادمغذی،ازنظرکمی وکیفی یکی ازاساسی ترین موادغذایی بوده‌‎و با توجه به شرایط مادران بارداروشیرده‌ یکی ازمناسبترین غذاها برای تأمین مناسب نیازهای این دوران بوده واثرات بسیار مفید و موثری بر مادر وجنین خواهدداشت. هدف از این مطالعه، بررسی آگاهی، نگرش و عملکرد مادران باردار در خصوص مصرف شیرولبنیات در شهر یزد است. روش بررسی:مطالعه حاضربه صورت توصیفی وازنوع مقطعی بوده‌که با مشارکت 254نفر از مادران شهر یزد در سال 1392 انجام شده‌است. ابزار جمع‌آوری اطلاعات پرسشنامه‌ای مشتمل بر متغیرهای دموگرافیک و سوالات آگاهی،نگرش وعملکرد بود. داده‌های جمع‌آوری شده‌ با استفاده ازنرم افزار 18SPSS وآزمون‌های توصیفی واستنباطی تجزیه وتحلیل شدند. یافته‌ها:میانگین سن مادران98/4 ±8/26سال بود،که 8/50% ازآنها،حاملگی اول‌شان راسپری می‌کردند. 8/39% از مادران در مورد شیر و لبنیات آگاهی خوب داشتند و 8/13% زنان از نگرش خوبی در این زمینه برخوردار بودند. در بخش عملکرد فقط 3/6% از مادران شیر و لبنیات را بر اساس مقادیرتوصیه شده مصرف می‌کردند. همچنین تفاوت معناداری بین مصرف شیردردوران غیربارداری وبارداری مشاهده‌شد. علاوه براین میان میزان تحصیلات و آگاهی رابطه معناداری نشان داده ‌شد. مطابق یافته‌های پژوهش آگاهی وعملکرد همبستگی معناداری نداشتند ولی همبستگی مستقیم ومعناداری میان نگرش، عملکرد وآگاهی، نگرش مشاهده ‌شد. نتیجه گیری:نتایج حاصل از این بررسی نشان داد که سطح آگاهی و نگرش اکثر مادران باردار درخصوص شیر و لبنیات در حد متوسط است و مقدار مصرف و لبنیات مادران باردار مورد مطالعه، کمتر از مقادیر توصیه شده می باشد. لذا برای ارتقای سلامتی مادران و فرزندان آنها که آینده ساز جامعه هستند نیاز به اتخاذ تدابیر لازم از جمله ارتقای آگاهی های تغذیه ای به خصوص در مورد مصرف شیر و لبنیات لازم است. این مقاله برگرفته از پایان نامه دانشجویی کارشناسی ارشد دانشگاه علوم پزشکی شهید صدوقی یزد می باشد.

Title: رنگزدایی از پساب واقعی نساجی با استفاده از فرایند انعقاد الکتریکی با الکترودهای آهن تک قطبی

http://tbj.ssu.ac.ir/article-1-1019-fa.html

مقدمه: رنگ یکی از شاخص های آلودگی آب می باشد و تخلیه پساب های رنگی می تواند به منابع آب های پذیرنده آسیب برساند. یکی از روش‌های پیشنهادی برای تصفیه فاضلاب رنگی استفاده از فرایند انعقاد الکتریکی می باشد. در مطالعه حاضر حذف رنگ از فاضلاب واقعی نساجی به وسیله فرایند انعقاد الکتریکی، با الکترودهای آهن تک قطبی مورد بررسی قرار گرفت. روش بررسی: این مطالعه در مقیاس آزمایشگاهی در یک راکتور الکتروشیمیایی ناپیوسته با حجم مفید 5/0 لیتر انجام شد. راکتور به 4 صفحه الکترود آهن مجهز بود که به حالت تک قطبی به یک منبع جریان برق مستقیم (DC) متصل بود. در هر مرحله از آزمایش ml 400 از پساب وارد راکتور شد و اثر پارامترهای عملیاتی دانسیته جریان ( mA/cm2 04/26- 2/5) ، pH (9-4) و زمان واکنش (min60-20) بر کارایی حذف رنگ و COD مطالعه شد. یافته‌ها: نتایج حاصل از مطالعه نشان داد که با افزایش دانسیته جریان، زمان واکنش وpH ، کارایی حذف رنگ و COD افزایش می یابد. شرایط عملیاتی بهینه در دانسیته جریان mA/cm262/15، زمان واکنش 60 دقیقه و 9=pH مشاهده گردید، که در آن کارایی حذف رنگ 92 و COD 89 درصد و مصرف انرژی kWh/m3 25/26 بود. نتیجه گیری: مطالعه حاضر نشان داد که فرایند انعقاد الکتریکی با الکترودهای آهن روشی مؤثر و کارآمد برای رنگزدایی پساب نساجی می باشد.

Title: رفتارهای پیشگیری کننده از عود سنگ کلیه و ارتباط آن با میزان آگاهی در این زمینه و منابع دریافت آن در بیماران

http://tbj.ssu.ac.ir/article-1-1020-fa.html

مقدمه: سنگ کلیه شایع ترین بیماری مزمن بعد از پر فشاری خون می باشد. عود این بیماری در جهان شایع بوده و تخمین زده می شود 50% سنگها، ظرف مدت 10 سال عود مجدد خواهند داشت. آگاهی بیماران از بیماری سنگ کلیه و روشهای پیشگیری از عود آن یک عامل مهم در انجام رفتارهای پیشگیری کننده از عود سنگ کلیه می باشد. در این مطالعه، رفتارهای پیشگیری از عود سنگ کلیه و ارتباط آن با میزان آگاهی و منابع کسب آگاهی مورد بررسی قرار گرفت. روش بررسی: این پژوهش یک مطالعه توصیفی-تحلیلی است که به صورت مقطعی انجام گردید. حجم نمونه مورد نظر شامل 210 نفر از افراد با سابقه سنگ کلیه مراجعه کننده به سه مرکز درمانی شهر یزد بود. ابزار جمع آوری اطلاعات، پرسشنامه ای شامل: بخش های مشخصات دموگرافیک و زمینه ای، ارزیابی آگاهی و راهنما برای عمل و رفتارهای پیشگیری کننده از عود سنگ کلیه بود که به صورت مصاحبه تکمیل می شد. داده ها وارد نرم افزار SPSS16 شده و جهت تحلیل آنها از آزمون های T-test، ANOVA، رگرسیون خطی، ضریب همبستگی پیرسون و آزمون 2χ استفاده شد. یافته ها: میانگین نمره رفتارهای پیشگیری کننده از عود 85/7±75/38 از 70، میانگین نمره آگاهی06/4±49/9 از 25 و میانگین نمره راهنما برای عمل 78/1±67/2 از 8 بود. در میان رفتارهای پیشگیری کننده از عود سنگ، کمترین فراوانی مربوط به مشورت با پزشک متخصص یا متخصص تغذیه درباره ی مصرف میوه و سبزی بود. آگاهی پاسخگویان، در موردچگونگی مصرف لبنیات در افراد دارای سابقه سنگ کلیه در پایین ترین سطح بود و بیشترین منبع کسب اطلاعات درمورد رفتارهای پیشگیری از عود، بیماران سنگ کلیه ای دیگر(3/54%) ذکر شد. آزمون همبستگی پیرسون بین آگاهی، راهنما برای عمل و رفتار پیشگیری کننده از عود سنگ کلیه همبستگی مثبت و معنا داری را نشان داد(05/0>p) . نتیجه گیری: با توجه سطح متوسط میزان آگاهی و عملکرد افراد تحت مطالعه و با توجه به بالا بودن سن افراد مبتلا به سنگ کلیه و همچنین سطح تحصیلات پایین در این گروه، کادر بهداشتی می تواند مهمترین منبع دریافت آگاهی این افراد درباره ی رفتارهای پیشگیری کننده از عود سنگ کلیه باشد. این مقاله حاصل پایان نامه دانشجویی دانشگاه علوم پزشکی شهید صدوقی یزد می باشد. .

Title: رابطه دلبستگی به خدا با معناداری زندگی والدین کودکان کم توان ذهنی شهر زاهدان

http://tbj.ssu.ac.ir/article-1-1024-fa.html

مقدمه: تولد کودک کم توان ذهنی کیفیت زندگی والدین را تحت تأثیر خود قرار می دهد و پذیرش واقعیت ها، شکیبایی و تحمل هنگام رویارویی با مسائل و مشکلات بستگی زیادی به جهان بینی والدین و چگونگی ارتباط شان با جهان هستی (معنویت) دارد. در همین راستا هدف مطالعه حاضر بررسی رابطه دلبستگی به خدا با معنادری زندگی والدین کودکان کم توان ذهنی شهر زاهدان می باشد. روش بررسی: مطالعه حاضر از نوع توصیفی- همبستگی است و جمعیت مورد مطالعه آن والدین کودکان کم توان ذهنی مراجعه کننده به مرکز بهزیستی شهر زاهدان در بین ماه های مهر تا آذر 1391 بود که به صورت نمونه گیری هدفمند 125 نفر به عنوان نمونه مورد مطالعه قرار گرفتند. برای جمع آوری اطلاعات از دو پرسشنامه دلبستگی به خدا (لارسن و غباری بناب، 2009) و مقیاس معناداری زندگی (باتیستا و آلموند،1973) استفاده شد. برای تجزیه و تحلیل داده ها از آزمون ضریب همبستگی اسپیرمن و رگرسیون چندگانه با استفاده از نرم افزار SPSS17استفاده شد. یافته ها: یافته ها نشان می دهد که سطح دلبستگی به خداوند و معناداری زندگی والدین کودکان کم توان ذهنی بالاتر از میانگین بود و بین دلبستگی به خدا و تمامی ابعاد آن با معناداری زندگی این گروه از والدین رابطه مثبت و معناداری وجود داشت. هم چنین از بین ابعاد دلبستگی به خدا، ابعاد جوارجویی، پایگاه امن، پناهگاه امن، ادراک مثبت از خود و اضطراب جدایی قابلیت تبیین و پیش بینی معناداری زندگی این گروه از والدین را داشتند. نتیجه گیری: با توجه به محرز شدن نقش دلبستگی به خدا در معناداری زندگی والدین کودکان کم توان ذهنی، تشکیل جلسات معنویت درمانی و آموزش استفاده از شیوه های مقابله مذهبی در حذف و بر طرف نمودن استرس ها و معنادارسازی زندگی این گروه از والدین توسط سازمان ها و نهادهای مربوطه از قبیل بهزیستی، آموزش و پرورش کودکان استثنایی و صدا و سیما پیشنهاد می گردد.

Title: نیازهای آموزشی زوجین جوان در زمینه بهداشت باروری در استان یزد

http://tbj.ssu.ac.ir/article-1-1606-fa.html

چکیده مقدمه: یکی از نیازهای اساسی زوجین جوان داشتن آگاهی در زمینه بهداشت باروری است که در بهبود سلامت و تحکیم بنیان خانواده تاثیر بسزایی خواهد داشت. شناخت نیازهای زوجین جوان توجه بیشتر برنامه ریزان را به مسائلی که زوجین کمتر از آن آگاهی دارند سوق می دهد ارائه آموزشهای مورد نیاز به زوجین جوان کمک می نماید تا افکار و احساسات خود را بهتر درک نموده و تصمیمات صحیح و مناسبتری در مورد موضوعات بهداشت باروری برای خود اتخاذ نمایند. روش بررسی: این مطالعه توصیفی تحلیلی می باشد و جامعه مورد پژوهش زوجین داوطلب مراجعه کننده به مراکز مشاوره قبل از ازدواج بودندکه یکسال از ازدواج آنها گذشته و نیازهای آموزشی خود را بعد از تجربه در زندگی بیان نمودند. نمونه ها شامل 240 زوج و ابزار جمع آوری داده ها پرسشنامه بود که بصورت مصاحبه تکمیل و اطلاعات با نرم افزار SPSS مورد تجزیه وتحلیل قرار گرفت. یافته ها: کم سوادها، روستائیان، شاغلین داخل منزل و کارگران،ازافرادی هستند که نیاز آموزشی آنها بیشتر از سایرین بود و از نظر آماری تفاوت معنی داراست(000/0 p=). اما نیاز آموزشی با سن ازدواج و جنس مرتبط نبوده و تفاوت معنی داری ندارد (05/0 p >). میزان نیاز آموزشی به موضوعات بهداشت دوران قاعدگی،چگونه باردار شدن، بیماریهای مقاربتی، بارداریهای پرخطر ، بیماریهای مادرزادی ،سرطا نهای شایع ،اختلالات جنسی را زیاد و خیلی زیاد بیان نمودند. نتیجه گیری: با توجه به نتایج مطالعه باید به نیازهای آموزشی زوجین توجه بیشتر نموده و برای گروه کم سواد و روستائیان کلاسهای تکمیلی بعد از ازدواج گذاشته شود و بر اساس نیازهای بدست آمده،سرفصلها برنامه‌ها و ا مکانات آموزشی مناسب با آنها را تدارک ببینند. واژه های کلیدی:زوجین جوان ،آگاهی، نیاز آموزشی

Title: مقایسه آزمایشگاهی میزان اثربخشی عصاره حنا، هیپوکلرید سدیم و نیستاتین در کنترل پلاک کاندیدا یی موجود بر روی قطعات رزین آکریل آلوده

http://tbj.ssu.ac.ir/article-1-1026-fa.html

مقدمه: استئوماتیت ناشی از دندان های مصنوعی یکی از مشکلات رایج در استفاده کنندگان از دندان های مصنوعی می باشد. این پدیده، ناشی از چسبندگی وکلونیزاسیون کاندیدا آلبیکنس بر روی آن می باشد. ضد عفونی کردن پروتز با ضدعفونی کننده های شیمیایی رایج می تواند منجر به ضایعات آلرژیک دهانی گردد. هدف از انجام این مطالعه مقایسه اثر بخشی عصاره آبی حنا و هیپوکلریت سدیم جهت ضد عفونی قطعات رزین آکریل که به صورت تجربی به کاندیدا آلبیکانس آلوده شده اند، می باشد. روش بررسی: در این مطالعه تجربی ابتدا تعداد 100 قطعه مربعی شکل به ابعاد mm 1× 10×10 رزین آکریل تهیه و سپس برای تشکیل پلاک تجربی کاندیدایی با سوسپانسیون کاندیدا آلبیکنس آلوده شدند. میانگین تعداد سلولهای کاندیدا متصل به قطعات رزین قبل از انجام ضدعفونی 12 قطعه پلاک تعیین گردید. سپس 88 پلاک آلوده به چهار گروه تقسیم و هر گروه به ترتیب به مدت 8 ساعت در mg/ cc 5 عصاره ی آبی حنا، سدیم هیپوکلریت 2% (گروه تست)، محلول 100000 واحدی نیستاتین (کنترل مثبت)، و آب مقطر استریل (کنترل منفی) به صورت غوطه وری بر روی شیکر در حرارت Cº37 قرار گرفتند. میزان پایداری کاندیدا با کشت محلول شستشوی قطعات آکریل بر روی محیط سابورو و مقایسه میانگین کلنیهای کاندیدا باقیمانده بر روی قطعات با کمک آزمونهای کروسکال والیس و من ویتنی با نرم افزار آماری SPSS با هم مقایسه شد. یافته ها: با مقایسه میانگین کاهش تعداد کلنی کاندیدا جدا شده از قطعات آکریل قبل و بعد از ضد عفونی با مواد مورد بررسی، این تفاوت برای پلاکهای رزین آکریل ضد عفونی شده با عصاره آبی حنا، سدیم هیپوکلرید، و نیستاتین معنی دار بود (0001/0=(P. در این مطالعه محلول سدیم هیپوکلرید به میزان 100% در حالیکه محلول عصاره آبی رقیق حنا توان کاهش بیش از 80% بیوفیلم کاندیدیایی تجربی روی قطعات آکریل داشت. آب مقطر استریل تنها با کاهش 7/24% کمترین اثر در کاهش میزان کلونیزاسیون کاندیدا برروی قطعات آکریل را نشان داد. نتیجه گیری: نتایج بدست آمده نشان می دهدکه که با توجه به ارزان بودن و همچنین نداشتن عوارض، می توان غوطه وری دست دندان در محلول رقیق عصاره حنا را جهت کنترل بیوفیلم کاندیدا و جلوگیری از استئوماتیت ناشی از دندان مصنوعی پیشنهاد کرد.

Title: بررسی تعداد بهینه نیروی پرستاری مورد نیاز بخش اورژانس مرکز آموزشی درمانی شفا کرمان

http://tbj.ssu.ac.ir/article-1-1027-fa.html

چکیده مقدمه: بخش اورژانس، قلب بیمارستان را تشکیل می دهد و نیروی انسانی متخصص منبع اصلی تولید و ارائه خدمت در این بخش می باشد که در هیچ شرایطی این بخش نباید با کمبود نیروی انسانی مواجه باشد، لذا استاندارد نمودن تعداد و نحوه توزیع نیروهای پرستاری در این بخش لازمه ارتقای کارایی و کیفیت خدمات ارائه شده به بیماران و ارتقای بهره وری در بیمارستانها است. روش بررسی: پژوهش حاضر از نوع توصیفی- تحلیلی بوده و جامعه پژوهش آن شامل پرونده های بیماران مراجعه کننده به بخش اورژانس بیمارستان مورد مطالعه می باشد. با استفاده از روش نمونه گیری خوشه ای، ماه مرداد به عنوان خوشه انتخاب گردید. تعداد نمونه ها با استفاده از فرمول تعیین حجم نمونه بدست آمد و 384 پرونده از ماه مرداد به صورت تصادفی انتخاب و مورد بررسی قرارگرفت. ابزار جمع آوری داده ها، چک لیست زمان سنجی خدمات پرستاری بود. به منظور تهیه چک لیست، پرونده های بیماران بخش اورژانس جهت شناسایی خدمات پرستاری در این بخش مورد بررسی قرارگرفت و 19 خدمت پرستاری لیست شد. چک لیست اولیه جهت اظهارنظر در اختیار مسئولان پرستاری بیمارستان و اساتید دانشگاه قرارگرفت و پس از اعمال تغییرات مورد نظر آنان، روایی آن تأیید گردید. جهت تحلیل داده ها از تکنیک برنامه ریزی خطی و نرم افزار12 Lingo استفاده گردید. یافته ها: از متوسط ورود بیماران، خدمات پرستاری ارائه شده در بخش و درصد بیمارانی که آن خدمات را دریافت کرده اند، به عنوان ورودی های مدل استفاده گردید. محدودیت های مدل عبارت بودند از محدودیت های دوره زمانی، محدودیت عرضه و محدودیت های غیر منفی بودن متغیرها. تحلیل خروجی های نرم افزار نشان داد تعداد بهینه پرستار در شیف صبح 14، عصر12 و در شیفت شب 15 نفر و در مجموع 42 نفر می باشد؛ لذا این بخش با 19 نفر کمبود پرستار بویژه در شیفت شب مواجه است. نتیجه گیری: تحلیل یافته ها نشان داد، بخش اورژانس بیمارستان مورد بررسی با کمبود پرستار بویژه در شیفت شب مواجه است. راه حلهای اجرایی جهت ایجاد تناسب بین تعداد پرستار و تعداد بیمار در این بخش را می توان در دو حوزه عرضه و تقاضای خدمات اورژانسی در ساعات مختلف شبانه روز، مورد بررسی قرارداد. از آنجا که تنها ساعات اولیه شیفت شب بخش با تعداد زیاد مراجعه کننده مواجه است؛ تخصیص منطقی اضافه کاری به پرسنل پرستاری شیفت عصر می تواند راهگشا باشد. همچنین باوجود آنکه مدیریت تقاضا برای خدمات اورژانسی با محدودیت های جدی مواجه است، بیمارستانها می توانند با اجرای صحیح پرستاری تریاژ، به گروه بندی بیماران از لحاظ اولویت دریافت خدمات بپردازند.

Title: اولویت بندی روش های کنترل وکاهش آلودگی صدا در شرکت سیمان لارستان با استفاده از فرایند تجزیه و تحلیل سلسله مراتبی(AHP)

http://tbj.ssu.ac.ir/article-1-1028-fa.html

مقدمه: مواجهه با آلودگی صدا عوارض مختلفی را در انسان برجای می گذارد. هدف ما در این مقاله اولویت بندی روش های کنترل و کاهش آلودگی صدا در شرکت سیمان لارستان با استفاده از فرایند تجزیه و تحلیل سلسله مراتبی است. روش بررسی: جهت غربالگری معیارها و روش های مورد استفاده درروش تجزیه و تحلیل سلسله مراتبی از روش دلفی استفاده شد. که پس از نظرخواهی از 15 کارشناس خبره ، 8 معیار و 9 گزینه با اجماع نظر آنان انتخاب گردید. سپس جهت اولویت بندی روش های کنترل و کاهش الودگی صدا با استفاده از روش تجزیه و تحلیل سلسله مراتبی به مقایسه زوجی هر گزینه به همراه معیار های آن توسط کارشناسان پرداخته شد . یافته ها: نتایج مقایسه زوجی معیار ها نشان داد که معیار هزینه سرمایه گذاری اولیه با وزن نسبی 247/. دارای بیشترین اهمیت و معیار میزان رضایتمندی از بکارگیری از روش، با وزن نسبی 035/. کمترین اهمیت را به خود اختصاص داد. همچنین نتایج مقایسه زوجی گزینه ها نسبت به هدف انتخاب روش کنترلی مناسب نشان داد که گزینه کنترل زمان مواجهه فردی با سرو صدا با وزن نهایی 224/0 اولویت اول و گزینه عایق بندی ساختمان ها با وزن نهایی 067/0 اولویت آخر را به خود اختصاص داد . بحث و نتیجه گیری: از آنجا که روش کنترل زمان مواجهه فردی با سرو صدا به عنوان بهترین روش کنترل آلودگی صدا در این شرکت بدست آمد، دستورالعمل ACGIH در خصوص میزان زمان مواجهه با سرو صدا در محیط کار به مدیریت پیشنهاد گردید.

Title: بررسی کارایی خاکستر هسته زیتون در حذف نیترات از محلول های آبی

http://tbj.ssu.ac.ir/article-1-1029-fa.html

مقدمه: حضور نیترات با غلظت بالا در آب آشامیدنی مخاطرات بهداشتی به همراه دارد. نیترات در معده یا بزاق به نیتریت احیا شده و بنابراین باعث ایجاد بیماری متهموگلوبینیا در نوزادان می گردد. هدف از این مطالعه بررسی کارایی حذف نیترات به وسیله خاکستر هسته زیتون از محلول های آّبی می باشد. روش بررسی: این مطالعه از نوع تجربی_ بنیادی می باشد که در مقیاس آزمایشگاهی و به صورت ناپیوسته انجام گرفته است. در این تحقیق خاکستر هسته زیتون در مجاورت با غلظت های مختلفی از محلول نیترات (mg/L 150، 100، 50 ) با pH (9، 7، 5، 3) و زمان ماند (180، 120، 60، 30،15 دقیقه)، وزن جاذب (8/0، 6/0، 4/0 gr) قرار گرفته و غلظت نیترات با استفاده از دستگاه اسپکتروفتومتر در دو طول موج 220 و 275 نانومتر اندازه گیری شد. یافته ها: نتایج حاصل از این تحقیق نشان داد که با افزایش زمان ماند و همچنین افزایش دوز جاذب میزان حذف افزایش یافته و رابطه مستقیمی بین این دو عامل وجود دارد و با کاهش مقدار pH و غلظت اولیه نیترات، مقدار راندمان حذف افزایش قابل توجهی می یابد. بررسی اثر غلظت اولیه نیترات در حذف نشان داد که با افزایش غلظت اولیه نیترات از 50 به 150 میلی گرم در لیتر با ثابت نگه داشتن دوز جاذب، راندمان جذب از 88% به 48% کاهش می یابد ولی ظرفیت جذب (qe) از 31/8 به 3/ 14 میلی گرم بر گرم افزایش می یابد. همچنین بیشترین میزان جذب نیترات در 60 دقیقه اولیه واکنش اتفاق افتاد. نتیجه گیری: بر اساس نتایج این تحقیق خاکستر هسته زیتون قادر به جذب نیترات در حد مطلوبی بوده و کارایی فرایند در pH اسیدی بیشتر می باشد. همچنین با توجه به نتایج به دست آمده از این تحقیق در مقایسه با روش های دیگر جهت حذف نیترات، روش فوق می تواند به عنوان روشی موثر و مناسب برای حذف نیترات مورد استفاده قرار گیرد.
